# Implications of Lifestyle and Occupational Factors on the Risk of Breast Cancer in Shiftwork Nurses

**DOI:** 10.3390/healthcare9060649

**Published:** 2021-05-30

**Authors:** Javier Fagundo-Rivera, Regina Allande-Cussó, Mónica Ortega-Moreno, Juan Jesús García-Iglesias, Adolfo Romero, Carlos Ruiz-Frutos, Juan Gómez-Salgado

**Affiliations:** 1 Health Sciences Doctorate School, University of Huelva, 21071 Huelva, Spain; javier.fagundo308@alu.uhu.es; 2 Centro Universitario de Enfermería Cruz Roja, University of Seville, 41009 Seville, Spain; 3 Escola Superior de Saúde, Universidade Atlântica, 2730-036 Barcarena, Portugal; 4 Department of Nursing, University of Sevilla, 41009 Sevilla, Spain; rallande@us.es; 5 Department of Economy, Faculty of Labour Sciences, University of Huelva, 21007 Huelva, Spain; ortegamo@uhu.es; 6 Department of Sociology, Social Work and Public Health, Faculty of Labour Sciences, University of Huelva, 21007 Huelva, Spain; frutos@uhu.es (C.R.-F.); jgsalgad@gmail.com (J.G.-S.); 7 Nursing and Podiatry Department, Health Sciences School, University of Malaga, Instituto de Investigación Biomédica de Málaga (IBIMA), 29071 Málaga, Spain; 8 Safety and Health Postgraduate Programme, Espíritu Santo University, Guayaquil 092301, Ecuador

**Keywords:** breast cancer, night work, shift work, health personnel, occupational disease, working conditions, prevention, carcinogens

## Abstract

Shift work that involves circadian disruption has been highlighted as a likely carcinogenic factor for breast cancer in humans. Also, unhealthy lifestyle habits observed in night work nurses could be causally related to an increase in the incidence of estrogen-positive breast tumours in this population. Assessing baseline risk of breast cancer in nurses is essential. The objective of this study was to analyze the risk of breast cancer that nurses had in relation to their lifestyle and labour factors related to shift work. A cross-sectional descriptive study through a questionnaire about sociodemographic variables, self-perception of health, and working life was designed. The sample consisted of 966 nurses. The relationship between variables was tested. A binary logistic regression and a classification and regression tree were performed. The most significant labour variables in relation to the risk of breast cancer were the number of years worked (more than 16 years; *p* < 0.01; OR = 8.733, 95% CI = 2.811, 27.134) and the total years performing more than 3 nights per month (10 or more years; *p* < 0.05; OR = 2.294, 95% CI = 1.008, 5.220). Also, the nights worked throughout life (over 500; OR = 4.190, 95% CI = 2.118, 8.287) were significant in the analysis. Nurses who had or ever had breast cancer valued their self-perceived health more negatively (*p* < 0.001) and referred a lower quality of sleep (*p* < 0.001) than the non-cases nurses. The occupational factors derived from night work could have several impacts on nurses’ health and their family-work balance. Promoting healthy lifestyles, informing about shift work risks, and adjusting shift work schedules are critical methods to decrease the possible effects of circadian disruption in nurses.

## 1. Introduction

Shift work, including night shift work, has been associated with circadian disruption in several epidemiological studies conducted on nurses [1,2,3,4,5] and in several targeted investigations [6,7,8,9] in which expression, methylation and polymorphisms of circadian genes that could be associated with breast cancer risk among shift nurses have been studied.

In fact, long-term rotating shifts (i.e., 12 h rotating shifts) and night work have been linked to the presence of tumours with oestrogen-positive (ER+) and progesterone (PR+) receptors [4,10,11,12,13,14,15], and to the luminal subtypes of tumour classification (mainly luminal A) in several studies using nursing populations [16,17]. In addition, high levels of oestradiol have been recorded in nurses performing night shifts compared to those performing day shifts [10,11,13], and several studies have found significant differences between nurses working on permanent night shifts and those who performed rotating shifts, concluding that long-term, high-intensity shifts for several consecutive years can significantly influence the risk of breast cancer. Under this evidence, shift work and night work were classified as likely carcinogenic factors (Group 2A) by the International Agency for Research on Cancer (IARC), especially incidents in those professions that, such as nursing, must adjust their work throughout 24 h a day [18,19,20,21,22,23], although in-depth studies confirming these findings are still required.

One of the most noted causes has been related to the loss of synchronisation between the circadian rhythm and the sleeping patterns of nurses during continuous and long-duration rotating shift work [18,19,24,25,26]. Also, eating at night or referring hunger during hours that are normally dedicated to nocturnal rest may lead to disturbances in the suprachiasmatic nucleus control of hunger and satiety cycles, intrinsically related to metabolic regulation mechanisms and energy activity in peripheral tissues [24]. In addition, certain levels of exposure to blue or artificial light at night are known to affect the circadian system, altering production times and melatonin levels, among other hormones [27,28].

Circadian and sleep disturbances (i.e., social jet lag [29] or shift work disorder [30]) can lead nurses to experience extreme tiredness [31,32], to have a less active life during free time [33], and to carry out poor dietary control [23], factors that together increase cardiovascular and diabetes risk [13,34,35,36,37], and pose an increased risk of breast cancer [18]. Thus, the duration of the work shift becomes a significant predictor of the quality of nurse care and job safety [38,39], since a longer duration of shifts has been linked to an increase in errors during attendance [40,41]. Therefore, occupational health specialists have the role of providing advice to managers and workers on the best strategies to reduce the negative effects of shift-induced circadian desynchronisation, by evaluating clinical symptoms and behaviours related to sleep-wake patterns, obesity, type 2 diabetes, or dyslipidaemia caused by the shift work disorder [24].

In view of the above, it is necessary to examine the risk profile of breast cancer that nurses working on rotating and night shifts have, as well as their perception of their own health and the factors that can harm or protect it. With this information, conclusions could be drawn that could facilitate health services’ managers’ decision-making when planning more appropriate work shifts to reduce risk factors of occupational breast cancer. In this way, the objective of this study was to analyse the risk of breast cancer and the self-perception of health by nurses in relation to lifestyle and occupational variables associated with shift work (including night shift).

## 2. Materials and Methods

### 2.1. Design and Sample

Cross-sectional, questionnaire-based descriptive study on the population of Registered Nurses in Spain, both men and women (currently 316,094 subjects) [42]. The sample selection was made by non-probabilistic snowball sampling, estimating the optimal size at 980 nurses with a 95% confidence level, 3.5% accuracy, and 20% adjustment for losses.

The inclusion criteria were (a) Being a nurse and being registered in the General Council of Nurses of Spain; (b) Working in private and/or public centres; (c) Wish to participate, having read and signed the informed consent. On the other hand, the exclusion criteria were (a) Not being Registered Nurses; (b) Being under 18; (c) Working outside Spain.

### 2.2. Shift Work and Night Work Definition

Cambridge Business English Dictionary defines shift work as: “a system in which different groups of workers work somewhere at different times of the day and night”. Besides, the IARC defined night work as: “one that requires at least three hours of work between midnight and 5 a.m.” [20,21,43]. The main characteristics of shift work are shown in Table S1 [11,20,21,23,44].

### 2.3. Instrument

To gather the appropriate information, an online questionnaire was used. The main risk variables related to breast cancer and night work which were used in this investigation were extracted and adapted ad hoc based on other questionnaires found in the scientific evidence [3,11,14,45,46]. The final questionnaire was formed by the 8 sections detailed below. The validation of the final instrument was carried out through two rounds of analysis by a panel of experts using a Delphi technique to determine whether the detected variables and the design of the questionnaire were relevant and appropriate in the context of the study. This group was made up of three nurses, two physicians, two psychologists, two members of a healthcare system management board and one methodologist. Subsequently, a piloting was carried out favourably in 10 people from different nursing areas to assess the suitability of the questions, possible grammatical errors or mistakes that were not previously detected.

### 2.4. Variables

The variables considered in this study were:

- Sociodemographic data (age, sex and marital relationship).
- Self-perception of health. An ad hoc evaluation tool consisting of 5 direct questions was designed to assess nurses’ perception of their own health. The valuation scale was a Likert type with ten response possibilities ranging from 1 “very low” to 10 “very high”. The questions used were: “How do you value your overall health?”, “How do you value the quality of your rest?”, “How do you value the effect shift work has on your health?”, “How do you value your level of work stress?” and “How do you value your satisfaction with your current job?”.
- General data on disease and cancer (current disease, oncological disease, number of mammograms, use of oral contraceptives and presence of first-degree familial cancer).
- Lifestyle habits (Body Mass Index-BMI-was calculated with the weight and height indicated by the participants. Working exertion, measured by light, moderate, hard or very hard. Free-time physical activity, measured by the time spent in hours).
- Family burdens (care for children under 14, and care for dependents or elderly family members at home).
- Sleeping aids (“Do you take any medication to sleep?”, “which?”).
- Exposure to tobacco (consumption habits (did you ever smoked?), exposure to tobacco in the workplace and at home).
- Labour information (type of current working schedule, working experience (throughout life), number of years working regularly 3 nights per month or more, number of worked nights accumulated throughout life, and sick leaves throughout life and in the last year).

### 2.5. Data Collection Procedure

The study development took place from December 2019 to November 2020. Google Forms© (Google, Mountain View, CA, USA) was used to create the online questionnaire. Participants could not access the questionnaire until they had previously done the following: (a) Having read and understood an introductory letter to the study and its objectives; (b) Having confirmed voluntary and anonymous participation in the study; (c) Declaring working as a nurse in Spain and being currently registered. The data obtained from the questionnaires were entered in Excel (Microsoft©, Redmond, WA, USA), R Commander 4.0.0 [47], and SPSS version 26.0 (IBM©, Armonk, NY, USA) for the statistical analysis.

The online questionnaire link was provided via email to the nurses who were registered in the General Nursing Council of Spain and previously had accepted to receive emails, news and information bulletins from this institution. The General Council of Official Nursing Colleges of Spain is the only regulatory body and competent authority of the nursing profession in Spain, therefore the registration in this government-authorized licensing body is obligatory to obtain a nursing license and be legally authorized to work, thus being considered a Registered Nurse (RN). Given this fact, a high number of nurses were consulted via email thanks to this collaboration, although we were not able to control this intervention due to the Data Protection policy of this institution. The social networks of official entities and professional groups of renowned prestige in the area of nursing in Spain (i.e., University of Huelva or Spanish Nurses Syndicate) also collaborated with the dissemination of the questionnaire.

### 2.6. Statistical Analysis

Absolute frequencies, percentages, and measures of position and dispersion, depending on the type of variable, described the variables of interest. Student’s T-test for independent samples and the Chi-squared test were used to contrast differences and relationship (OR) between the variables and breast cancer risk. The Mann–Whitney U nonparametric test for independent samples was used to analyse heterogeneity in the self-perception of health category.

Binary logistic regression allowed building a model to study the presence of breast cancer and identify those risk variables that played a relevant role. The Hosmer-Lemeshow test was used, and Odds Ratios (OR) were estimated with their confidence intervals.

**Figure 1:**
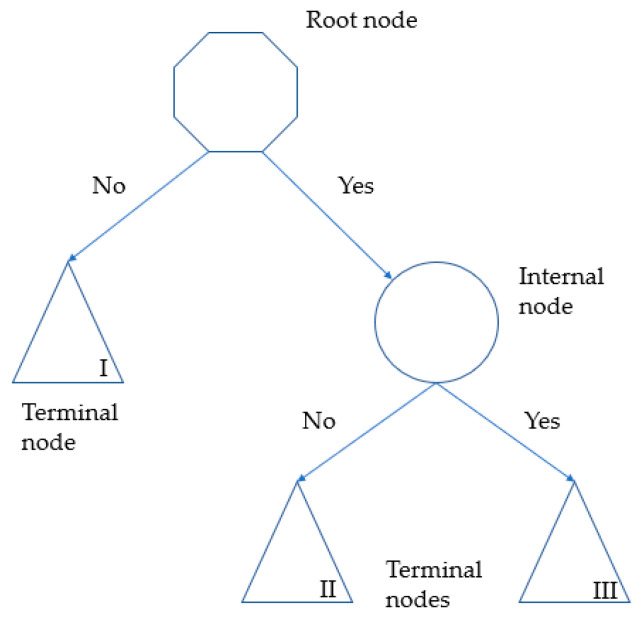
Decision Tree Classifier (for learning purpose).

Finally, the CART (Classification and Regression Trees) data mining method [48,49] was used to design a binary algorithm to predict which variables of the self-perception of health category played a significant role in breast cancer. The CART methodology refers to a model where the target variable and the algorithm itself are used to predict values based on several categorical or continuous input variables. It is shown as a Decision Tree Classifier (Figure 1), where each round is known as Node. Each node will have a question or if-clause according to which the subjects of study will be routed to a specific internal or terminal/leaf-node that will tell the final prediction. Each node shows the predicted class, the predicted probability of cases within the node, and the percentage of cases of the node over the total sample. In summary, there are three types of nodes:

- Root Node: represents a single input variable and a split point on that variable. It does not have any parent node and gives two children nodes based on the question.
- Internal Node: it will have a parent node and gives two children nodes.
- Terminal or Leaf Node: contains an output variable which is used to make a prediction. This node will have a parent node but will not have any children nodes.

### 2.7. Ethical Considerations

For this study, the Declaration of Helsinki (Fortaleza, 2013) was taken into consideration and explicit written permission was obtained from participants through their informed consent. Data obtained in this study were to be duly guarded by the research team, ensuring the confidential use and processing in accordance with the Organic Law on Protection of Personal Data and the Guarantee of Digital Rights. Approval was obtained from the Research Ethics Committee of the province of Huelva, belonging to the Regional Government of Andalusia, with code TD-CMTE-2020. Likewise, approval was obtained from the Research Ethics Committee of the General Council of Official Nursing Colleges of Spain with code PI 2109/02/CE.

## 3. Results

### 3.1. Two-Dimensional Analysis for Healthy Participants and Those Who Have or Ever Had Breast Cancer

The questionnaire was answered by a total of 966 nursing professionals, of whom 502 (51.97%) were healthy, 99 (10.25%) had or ever had some form of cancer, and 365 (37.78%) had another type of disease. Of those who had or ever had cancer, for 56.57%, it was breast cancer (56 subjects). Common diseases found in both men and women were hypertension, diabetes, obesity, asthma, or seasonal allergy. In women, isolated cases of thyroid cancer, cervical cancer, melanoma, leukaemia, and lymphoma were also found. 

For the bivariate analysis, healthy individuals (502) and breast cancer patients (56) were compared with the variables of interest. The median age of the sample was 41 years (*p* = 0.367) and 89.6% of the sample were women (*p* = 0.705). Subsequent bivariate analysis categorized by sex revealed only age (χ^2^ = 9.669; *p* 0.002; OR = 2.466; 95%CI= 1.376, 4.419) as significant in relation to breast cancer (see Table S2). According to the current workplace, a 19.2% of the sample worked on permanent shifts, mostly morning shifts, and a 74.5% worked on rotating shifts. Only a 3.8% worked on 24h-shifts. Approximately a 72.4% of the sample was working on night shifts at the time of the survey (Table 1).

No statistically significant differences were found for parity, either in the general (*p* = 0.684) and sex-categorized analysis (*p* = 0.648). On the other hand, significant associations with breast cancer were found in participants with a partner (*p* = 0.041) and those who cared for dependents at home outside the working hours (*p* < 0.001). Indeed, being single (OR = 0.541; 95% CI = 0.298, 0.982) and without family caring responsibilities (OR = 0.288; 95% CI = 0.146, 0.569) were related with breast cancer risk reduction (Table 2).

Having ever had a mammogram and the number of performed mammograms also resulted significative for breast cancer risk (mean = 2.27; SD = 4.43; *p* < 0.001; OR = 1.353, 95% CI = 1.248, 1.466). Similarly, the presence of familial cancer was significant (*p* = 0.005), resulting in risk reduction when there was no family cancer history (OR = 0.398; 95% CI = 0.205, 0.773) (Table 2).

In terms of lifestyle habits, BMI showed significant differences (*p* = 0.045). The higher number of breast cancer cases had normal BMI. Similarly, statistically significant differences (*p* < 0.001) were detected depending on the intensity of physical activity at work. Most breast cancer cases classified their exertion at work as moderate. However, no significant differences were found for the physical activity during free time (*p* = 0.631). Statistically significant differences were found regarding the frequency of exposure to tobacco smoke at home (*p* = 0.001) and the compliance with the smoking ban in the workplace (*p* = 0.010), but not with having ever smoked (*p* = 0.953). 

Lastly, the 79.2% of the nurses claimed not to take any sleep medication, although this variable was relevant in breast cancer cases (*p* < 0.001). In fact, not taking sleep medications resulted in risk reduction (OR = 0.138; 95% CI = 0.077, 0.247) (Table 2). The most common medication was oral melatonin (60 subjects, of which 11 had breast cancer). Among other remedies, infusions (valerian, melissa), hypnotics (doxylamine, zolpidem), anxiolytics (alprazolam, bromazepam), antidepressants (trazodone), and other benzodiazepines (lorazepam, lormetazepam, diazepam) were used. Oral contraceptives were only used by women in this study, not being significant for breast cancer (*p* = 0.441).

With respect to labour data (Table 3), being not currently working at night (*p* < 0.001; OR = 2.708, 95% CI = 1.548, 4.735) and being not currently shift working (*p* < 0.001; OR = 3.148, 95% CI = 1.765, 5.615) showed a statistical association with the risk of breast cancer. Considering the working history of the participating subjects, having exceeded 16 years of work was presented as one of the most significant variables in this study (*p* < 0.001; OR = 12.346, 95% CI = 4.854, 31.250). Nurses who had or ever had breast cancer had worked a mean of 26.1 years (SD = 8.1), while healthy nurses had worked a mean of 15.0 years (SD = 9.2). On the other hand, the percentage of cases with breast cancer was also higher in professionals with 500 or more nights worked (OR = 4.190, 95% CI = 2.118, 8.287) and when more than 3 nights per month had been worked for more than 10 years (OR = 4.132; 95% CI = 2.227, 7.634), finding a statistically significant difference in both situations (*p* < 0.001). The mean number of nights worked was 627.9 (SD = 639.4) in the case of healthy subjects and 1017.4 nights in those who had or ever had breast cancer (SD = 837.9). 2.3% of respondents never worked night shifts. Finally, the cumulative number of sick leaves, both in the last year (mean 0.35; SD = 0.542) and throughout the professional life (mean 2.20; SD = 2.118), have shown a statistically significant association with breast cancer (*p* < 0.001 in all cases) (Table 3).

### 3.2. Self-Perception of Health Descriptive Analysis

Regarding the self-perception of health, the overall health was rated 7.94 (SD = 1.26) among the sample nurses, being lower in breast cancer cases (6.45) and higher in the healthy cases (8.11). The lowest value of this category was found in the quality of sleep and rest, with a mean of 6.28 (SD = 1.96) and decreasing to 5.29 in breast cancer cases. The highest value was identified when considering whether shifts affect the health of people, with a mean of 9.08 (SD = 1.37). In relation to stress at work, the mean value was 7.57 (SD = 1.86). The stress perception was higher among breast cancer cases (8.23) as compared to healthy cases (7.49). Finally, the satisfaction with the working conditions was valued with a mean of 7.28 (SD = 1.87). Among all the health self-perception variables, statistical differences were found in terms of overall health (*p* < 0.001), sleep and rest quality (*p* < 0.001), and stress at work (*p* = 0.002) (Table 4).

### 3.3. Breast Cancer Prediction

The following two regression methods were performed in this study to identify those variables that play a relevant role in breast cancer risk.

#### 3.3.1. Considering Labour Variables and Sleep Medication

Binary logistic regression analysis predicts breast cancer among nurses through the following variables: total years performing more than 3 nights per month, sleep medication, sick leaves, years worked, and actual exposition to night work. This model was validated with the Hosmer-Lemeshov test (*p* = 0.811), correctly classifying 91.3% of cases. In addition, all variables included in this model had significant values lower than 0.05 and OR values greater than the unit (Table 5).

#### 3.3.2. Self-Perception of Health Variable

With regard to the self-perception of health among nurses, the CART showed the 558 cases at a root node, of which 10.03% (0.10; 56 subjects) have breast cancer. 

A second node differed according to the valuation of the overall health (higher or equal to 5.5; yes or no), resulting in a breast cancer percentage of 68% (0.68) in the 25 subjects (4% of the total cases) who valued their overall health below 5.5, and 7.3% of breast cancer cases in the subsequent 533 subjects (96% of the sample) whose scores were equal to or higher than 5.5. 

For the 4% of cases with worse health perception (overall health below 5.5), an internal node differed according to the satisfaction with the current working conditions. The percentage of breast cancer cases reached 82.4% (0.82) when the level of job satisfaction was higher or equal than 5.5, and otherwise the percentage of cases was 38% (0.38). 

Returning to those cases with overall health higher or equal to 5.5 (96%; 533 subjects), the following internal node differed again according to the valuation of the overall health (higher or equal to 7; yes or no). 502 nurses (90%) whose self-perception of health was ≥7 showed a 5.8% (0.06) of breast cancer cases. Otherwise, the 6% (33) of nurses whose self-perception of health was between 5.5 and 7 points comprised 29.4% (0.29) of cancer cases. 

**Figure 2:**
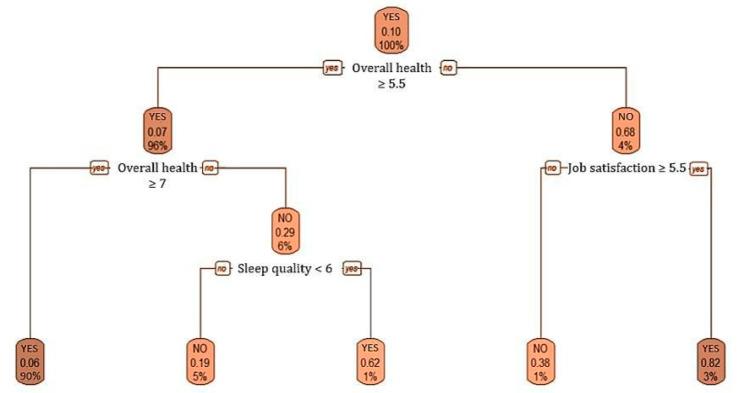
Classification and regression tree of breast cancer cases and self-perception of health.

Finally, an internal node for sleep quality (<6; yes or no) is shown for the cases with overall health between 5.5 and 7. 62% (0.62) of breast cancer cases occurred in 1% (6) of nurses who perceived their sleep quality under 6. On the other hand, 19% of breast cancer cases occurred in the 5% (28) of nurses who perceived their sleep quality over 6 (Figure 2).

## 4. Discussion

The main objective of this study was to analyse the relationship between shift work, especially night shift work, and the risk of developing breast cancer in nursing professionals in Spain, in order to obtain a descriptive image of the labour and lifestyle factors that can influence the risk of breast cancer in this group.

The logistic regression model was successfully validated and showed significant values. Among the measurements of night work that appear to be associated with the risk of breast cancer, having worked a mean of three night shifts per month for 10 years or more (OR = 2.294; 95% CI = 1.008, 5.220) has been highlighted, although the number of total years worked was also significant (OR = 8.733; 95% CI = 2.811, 27.134), taking as a reference the performance of professional activity for more than 16 years and that 95% of the sample reported having worked in rotating shifts at some point of his or her career, although at the time of the survey it was 80.8%. Also noteworthy in this study is the increased risk of breast cancer when 500 nights or more have been worked throughout life (*p* < 0.001; OR = 4.190; 95% CI = 2.118, 8.287). In accordance with other studies [36,50], the data provided suggests that the risk profile of shift-related breast cancer highly varies depending on the number of nights worked, so exposure to permanent and rotating night shifts is considered of key relevance from an early age and throughout the working life. In this way, several studies confirmed the risk of breast cancer among nurses working on rotating night shifts at least 3 nights a month for 20 years or more, particularly those who started in their young adulthood (before the age of 30) [3,12,16]. In premenopausal women, those characteristics of night work that were indicative of high intensity of exposure (3 or more nights per week), long duration of night work over life (at least 10 years in a row), and long night shifts (10 or more hours) were associated with an increased risk of breast cancer at 5 years of their working life [3,15,51,52]. 

On the other hand, the analysis of nurses’ self-perception of health variables and the CART classification and regression tree have made it possible to highlight the importance of attending to the assessment of general health, sleep quality, stress level, and level of job satisfaction referred to by nurses themselves. The present study has confirmed that nurses who had or ever had breast cancer exhibited a higher level of work-related stress and worse self-perceived health than healthy nurses. Equally, it is important to consider the impact of stress at home, as it can lead to work-family related conflicts and health problems [5,34,53,54]. In fact, the perception of stress in the family environment could be considered relevant given the positive association between the care of dependents at home (*p* < 0.001; OR = 0.288; 95% CI = 0.146, 0.569), having a partner (*p* = 0.041; OR = 0.541; 95% CI = 0.298, 0.982) and the risk of breast cancer found in this study. It is therefore worth noting that the work-family balance can affect the role of nurses [55] and the family stability [56,57], and this may lead to lack of time for leisure and self-care [5,58], tiredness [31], sleep problems [59] and many risk behaviours as alcohol consumption [53] if an important imbalance is given.

The risk profile analysis of nursing professionals in this study identified that professionals who had breast cancer valued worse the quality of their rest (5.29) than healthy professionals (6.39), with significant differences being detected between both groups (*p* < 0.001), although it is true that the quality of rest had a surprisingly low mean value between the two groups (6.28). Despite that, only 20% of respondents resorted to sleep medication, even though this variable showed a statistical significance for breast cancer in the model (OR = 5.841; 95% CI = 2.848, 11.978). Hypnotics intake has been associated with cancer due to the alterations on sleep patterns, such as insomnia, that occur during the disease at any stage and that persist in survivors [26,60]. In addition, slow rotating systems which include longer sequences of consecutive night shifts, can cause disturbances in sleep patterns [3,61], fatigue, and sleepiness even on rest days after shifts [62]. Other studies have shown that irregularity in the organization of the shifts affects the adaptive ability of shift work nurses [18,63,64]. 12-h shifts (day–night) imply less sleep disorders and a more balanced rest period than the 3 × 8 rotation (morning–afternoon–night) [18,65], thus allowing a better recovery. However, workload is more intense in 12-h shifts than in 3 × 8 shifts, due to the longer duration of the shift, resulting in greater physical and mental fatigue [18,64,65]. 

According to a recent study, when there are no symptoms of depression, anxiety, tiredness or stress, nurses are more resilient and more satisfied with their work [66]. In fact, job satisfaction is an interventional stress-reducing factor that can positively influence the perception of one’s own health [67,68], the decrease in the frequency of physical and psychological symptoms [69], and the improvement of the quality and safety of patient care [70,71,72], as well as the re-induction of errors [40]. Instead, low job satisfaction is a contributing factor to nurses leaving their jobs and profession [73,74,75]. Data from this study have shown a mean satisfaction of 7.28, that does not differ significantly between the assessed groups. This would make it possible to emphasise that job satisfaction is not one of the most prominent problems regarding the sample of this study since, as can be seen in the CART, low job satisfaction was not a criterion for pointing out a large number of cases of breast cancer.

Finally, according to data on lifestyle habits, physical activity in the working context was statistically significant in relation to breast cancer, reporting the majority of cases when it was classified as “moderate”. This may be contrary to other results that report a beneficial association of physically active jobs to reduce breast cancer risk [76]. Meanwhile, physical activity during free time was not significant in our study (*p* = 0.631). With reference to BMI, the 14.3% of the sample who had breast cancer were obese and the 30.4% had overweight. Obesity has been associated with consecutive night shifts (more than 8 shifts per month) and cumulative years of night work (more than 20 years) [36,77,78], as well as increased tobacco consumption [51]. In this line, recent research was consistent with the increased risk of breast cancer that occurs in active, heavy, and long duration smokers and passive smokers [51,79,80,81], particularly premenopausal women who smoked or were exposed to second-hand smoke between menarche and first full-term pregnancy, in the occupational and residential context [80]. This relationship could correspond to the results of the present study, which significantly linked breast cancer to tobacco exposure both in the workplace (*p* = 0.010) and at home (*p* = 0.001). 

It is worth noting that the responses to the questionnaire on current work have identified that not undertaking shifts nor night work were risk factors for breast cancer, given that most of breast cancer cases did not work on shifts by the time of the survey. These results were in line with a literature review on returning to work following a breast cancer process in Spain [82], in which it was concluded that improvements in working conditions allow workers to adapt their job to their new physical capacity. As described in another study [5], those individuals with breast cancer could have received a change or compensation on behalf of their workplace organisation when they were diagnosed or when re-joined after the sick leave, exempting them from rotating shifts and night work in order to create a less aggressive work environment for the worker. 

### 4.1. Implications for the Practice and Applicability

With a view to future research, investigating nurses’ breast cancer risk factors will still play an important role due to its importance in screening and prevention. Detailed information about nurses’ working hours and night work-related circadian disruption may also be relevant to increase visibility regarding this occupational health issue. In this sense, it would be interesting to measure total nocturnal output of melatonin in shift workers to make a correlation with total and nocturnal melatonin levels. Cortisol levels also require attention due to its balance with melatonin levels and regulation during day light. It must be noted that future research should also study specific parameters of circadian disruption, such as the expression of peripheral clock genes and clock-controlled genes. Periodic assessment of parameters such as waist circumference, waist-to-hip ratio, BMI, glycemia, glycosylated haemoglobin, triglycerides, and total HDL and LDL cholesterol would be useful to evaluate the risk of night shift-related diseases, such as obesity, type 2 diabetes, dyslipidaemia and metabolic syndrome [83]. 

It is appropriate to adopt prevention and action guidelines that highlight associations between diet, weight, and physical activity to prevent the development of breast cancer and possible metabolic syndrome on shift workers, taking into account cases in premenopausal and postmenopausal women whenever possible [84,85,86,87]. It has recently been shown that the adoption of dietary recommendations by Spanish women who have had cancer was moderate. At the same time, adherence to physical activity and body weight management was higher among older women, women who had one or more children, and those who lived in rural areas. Increased compliance with smoking bans and greater limitation of alcohol consumption were also reported [86].

The results obtained in this study may have applicability in different spheres. In the university education system, it would be relevant to talk about the risk of shift-based breast cancer and cardiovascular risk in order to raise awareness among students of the repercussions of intensive night work during the first years of the career and the risks associated with circadian disruption in order to prevent them.

For the management and human resources systems of healthcare companies, this study would allow managers and supervisors to consider an equitable distribution of shifts and breaks, as well as a limitation of weekly night shifts performed by nurses and overtime (extra-duties).

Similarly, for night workers in general, and for nurses in particular, this study should encourage interventions to promote a healthy and balanced life in order to counteract the negative effects of work on rotating and night shifts. In this way, workers should be informed of the potential risks of performing nights intensively or for several years, and it would be appropriate to provide balanced diets, to install suitable spaces and lighting for work [88] and rest, and to allow sufficient time to eat, pause, and organise work efficiently.

As for nurses who have or have had breast cancer, this study makes it possible to wonder whether shift-related breast cancer could be considered an occupational disease. In such case, it would be desirable to demonstrate individual risk by counting nights and analysing biomarkers. If possible, people who have had breast cancer should be encouraged to return to work through a process of time adaptation and activities.

### 4.2. Limitations

With regard to the limitations of the study, it should be kept in mind that it was a cross-sectional study and these results, although in line with previous evidence, must be considered with prudence to avoid interpretation bias. In addition, breast cancer diagnosis was not clinically confirmed because data was collected using self-reported information. It is also important to consider the recall bias as another study limitation related to the retrospective study design. In this sense, some variables such as weight, height or total number of worked nights could have been influenced by recall bias. On the other hand, our analysis could be influenced by a small sample size, as it covered only 56 cases of breast cancer, which could potentially represent participation bias although the sample and number of responses has been estimated as sufficient to overcome low representativeness. 

Many variables have not been considered in this study, such as menopausal status, age at menarche, age at first full-term parity, breastfeeding history, sleeping patterns or comorbidities. In this way, the authors encourage its detailed study in future investigations. 

Another limitation that has been perceived refers to precision of the intensity grades of the physical activity at work and self-perception of health. In this study, the exertion scores have been stated as light, moderate, hard or very hard, similarly to those scores found in the Borg Rating of Perceived Exertion Scale [89]. However, greater detail of the description provided on the scales should have been considered, according to various authors who proposed a detailed explanation [90,91]. Health-related quality of life and self-perception of health are commonly incorporated as predicting survival factors in the design of research studies and clinical trials in oncology. This study has used a self-assessment scale with a range from 1 to 10, which has allowed the CART method to be performed later. However, it should be noted that there are several validated tools for health assessment and quality of life, so their use should be considered in future research [92,93,94,95]. In this same line, this study did not use standard tests to assess the quality of sleep and the state of the circadian system (chronotype). Some scales are purposed like the Pittsburgh Sleep Quality Index [96] or the Morningness-Eveningness stability scale improved (MESSi) [97].

## 5. Conclusions

This study has highlighted a statistically significant increase in the risk of breast cancer in nurses in association with total years of work experience (the risk increases by over 16 years worked) and the total number of years of more than 3 nights per month (the risk increases by over 10 years worked). In addition, other factors such as the total number of nights worked (the risk increases by more than 500 nights), taking medication to sleep, and having had sick leaves have been associated. Similarly, certain variables related to health self-assessment, such as poor overall health quality, stress levels, or low sleep quality, have been significant in pointing out an increased risk of breast cancer.

Given the incidence of breast cancer in nurses and the frequency of night shift working further research is needed to clinically measure the possible effects of shift work on breast cancer risk, circadian misalignments and other metabolic diseases that could affect nursing professionals. However, many interventions could be developed from the actual moment in order to prevent and inform about this carcinogenic factor.

## Figures and Tables

**Table 1:** Description of the sample’s work organization.

	Cases	Percentage
Permanent Shift	107	19.2%
*Only morning*	73	68.2%
*Only afternoon/evening*	5	4.7%
*Only night*	29	27.1%
Rotative 3 Shifts/24 h Cycles (M/AE/N)	212	37.9%
Rotative 2 Shifts/24 h Cycles	204	36.6%
*Only morning + eventual extra-duty (+17 h)*	33	16.2%
*12-h shifts*	71	34.8%
*Rotative M and N*	31	15.2%
*Rotative M and AE*	62	30.4%
*Rotative AE and N*	7	3.4%
24-h Shifts	21	3.8%
Irregular	14	2.5%
Total general	558	100%

M: morning (7-h shift); AE: afternoon/evening (7-h shift); N: night (10-h shift).

**Table 2:** Sociodemographic and lifestyle characteristics of the sample.

	N (%)	Healthy Cases (%) (N = 502)	Breast Cancer Cases (%) (N = 56)	χ^2^	*p*	Odds Ratio (CI = 95%)
Sex						
Male	58 (10.4)	53 (10.6)	5 (8.9)	0.144	0.705	1.203 (0.460, 3.145)
Female	500 (89.6)	449 (89.4)	51 (91.1)			
Age						
41 or younger	281 (50.4)	256 (51.0)	25 (44.6)	0.813	0.367	1.290 (0.741, 2.247)
Older than 41	277 (49.6)	246 (49.0)	31 (55.4)			
Marital relationship						
With partner	317 (56.8)	278 (55.4)	39 (69.6)	4.178	0.041	0.541 (0.298, 0.982)
Single	241 (43.2)	224 (44.6)	17 (30.4)			
Children under 14 years						
Yes	225 (40.3)	201 (40.0)	24 (43.0)	0.166	0.684	0.890 (0.509, 1.558)
No	333 (59.7)	301 (60.0)	32 (57.1)			
Care for dependents at home						
Yes	58 (10.4)	44 (8.8)	14 (25.0)	14.257	<0.001	0.288 (0.146, 0.569)
No	500 (89.6)	458 (91.2)	42 (75.0)			
Mammography (N = 497)						
Yes	211 (42.5)	156 (35.3)	55 (100)	*	<0.001	1.353 (1.248, 1.466)
Never	286 (57.5)	286 (64.7)	0 (0)			
Family history of cancer (N = 551)						
Yes	72 (13.1)	58 (11.7)	14 (25.0)	7.814	0.005	0.398 (0.205, 0.773)
No	479 (86.9)	437 (88.3)	42 (75.0)			
BMI						
Underweight	10 (1.8)	8 (1.6)	2 (3.6)	8.074	0.045	-
Normal	376 (67.4)	347 (69.1)	29 (51.8)			
Overweight	128 (22.9)	111 (22.1)	17 (30.4)			
Obese	44 (7.9)	36 (7.2)	8 (14.3)			
Physical activity at work						
Light	124 (22.2)	114 (22.7)	10 (17.9)	30.175	<0.001	-
Moderate	313 (56.1)	283 (56.4)	30 (53.6)			
Hard	113 (20.3)	103 (20.5)	10 (17.9)			
Very hard	8 (1.4)	2 (0.4)	6 (10.7)			
Physical activity during leisure time						
Two hours or less	286 (51.25)	259 (51.6)	27 (48.2)	0.230	0.631	1.144 (0.659, 1.887)
More than 2 h	272 (28.75)	243 (48.4)	29 (51.8)			
Tobacco consumption						
Yes	301 (53.9)	271 (54.0)	30 (53.6)	0.003	0.953	1.016 (0.584, 1.770)
No	257 (46.1)	231 (46.0)	26 (46.4)			
Compliance with the smoking ban at work						
Totally	124 (22.2)	104 (20.7)	20 (35.7)	11.377	0.010	-
Almost always	239 (42.8)	213 (42.4)	26 (46.4)			
Hardly ever	141 (25.3)	132 (26.3)	9 (16.1)			
Never	54 (9.7)	53 (10.6)	1 (1.8)			
Exposure to tobacco smoke at home						
More than 5 h a day	22 (3.9)	15 (3.0)	7 (12.5)	15.967	0.001	-
Between 1 and 5 h a day	36 (6.5)	36 (7.2)	0 (0)			
Less than 1 h a day	42 (7.5)	39 (7.8)	3 (5.4)			
Never or hardly ever	458 (82.1)	412 (82.1)	46 (82.1)			
Use of medication to sleep						
Yes	116 (20.8)	83 (16.5)	33 (58.9)	54.988	<0.001	0.138 (0.077, 0.247)
No	442 (79.2)	419 (83.5)	23 (41.1)			
Hormone-based oral contraceptives (women only) (N = 504)						
Yes	334 (66.3)	295 (65.7)	39 (70.9)	0.594	0.441	0.786 (0.425, 1.451)
Never	170 (33.7)	154 (34.3)	16 (29.1)			

*: Fisher. BMI: Body Mass Index. Under 18.5: Underweight; (18.5, 25) Normal; (25, 29.9) Overweight; equal or higher to 30: Obese.

**Table 3:** Labour variables and risk of breast cancer.

	N (%)	Healthy Cases (%) (N = 502)	Breast Cancer Cases (%) (N = 56)	χ^2^	*p*	Odds Ratio (CI = 95%)
Shift work at this moment						
Yes	444 (79.6)	411 (81.9)	33 (58.9)	16.315	<0.001	3.148 (1.765, 5.615)
No	114 (20.4)	91 (18.1)	23 (41.1)			
Night work at this moment						
Yes	378 (67.7)	352 (70.1)	26 (46.4)	12.940	<0.001	2.708 (1.548, 4.735)
No	180 (32.3)	150 (29.9)	30 (53.6)			
Working experience						
16 years or less	280 (50.2)	275 (54.8)	5 (8.9)	42.369	<0.001	12.346 (4.854, 31.250)
More than 16 years	278 (49.8)	227 (45.2)	51 (91.1)			
Total years performing more than 3 nights a month						
10 years or less	317 (56.8)	302 (60.2)	15 (26.8)	22.870	<0.001	4.132 (2.227, 7.634)
More than 10 years	241 (43.2)	200 (39.8)	41 (73.2)			
Total worked nights						
Less than 500 nights	265 (47.5)	254 (50.6)	11 (19.6)	19.358	<0.001	4.190 (2.118, 8.287)
500 nights or more	293 (52.5)	248 (49.4)	45 (80.4)			
Total sick leaves over lifespan (N = 550)						
2 or less	342 (62.2)	329 (66.3)	13 (24.1)	36.977	<0.001	6.211 (3.236, 11.905)
More than 2	208 (37.8)	167 (33.7)	41 (75.9)			
Sick leaves in the last year (N = 554)						
Without sick leave	385 (69.5)	368 (73.6)	17 (31.4)	40.782	<0.001	6.061 (3.300, 11.111)
With sick leave	169 (30.5)	132 (26.4)	37 (68.5)			

**Table 4:** Sample profile according to the health self-perception variables.

From 1 to 10…	M (SD) (*N* = 558)	Breast Cancer Cases (*N* = 56)	Non Cases (*N* = 502)	Mann Whitney-U	*p*
*How do you value your overall health?*	7.94 (1.26)	6.45 (1.61)	8.11 (1.09)	5920.500	<0.001
*How do you value your sleeping* *quality?*	6.28 (1.96)	5.29 (2.06)	6.39 (1.91)	9741.500	<0.001
*How do you value the effect shift work has on your health?*	9.08 (1.37)	9.16 (1.60)	9.07 (1.35)	15,223.500	0.262
*How do you value your level of work stress?*	7.57 (1.86)	8.23 (1.67)	7.49 (1.87)	17,571.000	0.002
*How do you value your satisfaction with your current job?*	7.28 (1.87)	7.02 (2.09)	7.31 (1.85)	12,903.500	0.305

**Table 5:** Logistic regression analysis for breast cancer.

	Coefficient	OR	CI = 95% for OR	
			Inferior	Superior
Number of working years ^1^	2.167 **	8.733	2.811	27.134
Medication to sleep	1.765 **	5.841	2.848	11.978
Night work at this moment	1.701 **	5.479	2.520	11.915
Sick leave last year	1.684 **	5.387	2.527	11.484
Total years performing more than 3 nights per month ^2^	0.830 *	2.294	1.008	5.220
Constant	−1.814 **	0.163		
Sensitivity/Specificity		52.8%/95.9%		
Correctly classified percentage		91.3%		
R2 Cox and Snell/R2 Nagelkerke		0.228/0.461		
Hosmer-Lemeshov Test		0.811		
Omnibus test		< 0.001		

OR: Odds ratio. * *p* < 0.05; ** *p* < 0.01. ^1^: (more than 16 years); ^2^: (10 or more years).

## Data Availability

All generated data is presented within this paper and its Supplementary Material. The research team and the University of Huelva are responsible of keeping the datasets of the study, which would be available for investigators under reasonable query.

## Supplementary Materials

The following are available online at https://www.mdpi.com/article/10.3390/healthcare9060649/s1, Table S1: Shiftwork organization and characteristics; Table S2: Sex Analysis.
