# Supplementary material for: Implications of Lifestyle and Occupational Factors on the Risk of Breast Cancer in Shiftwork Nurses
Source: Healthcare (Basel). 2021 May 30;9(6):649. doi: 10.3390/healthcare9060649 (PMC8228409; doi:10.3390/healthcare9060649)
Supplement: Supplementary file 1 [file healthcare-09-00649-s001.zip › Table S1. Shiftwork data.pdf]

Table S1. Shiftwork organization and characteristics [26,27,44,55,70]

**Shiftwork organization**	
Permanent	People work regularly on one shift only, i.e., morning or afternoon or night.
Daytime work	Morning shift (08:00 AM – 03:00PM), afternoon/evening shift (03:00PM - 10:00 PM).
Split shift	Working time in two periods each day (i.e., 4 hours in the morning and 4 hours at night).
Night work*	In Spain, from 10:00 PM – 08:00 AM.
Rotating	People alternate working periodically on different shifts, always including nights (morning/night, afternoon/night, or morning/afternoon/night).
3-shift rotation	24 hours divided into three 8-hour shifts. The day is divided into three working periods: morning, afternoon and night.
6x2 fast rotation	Two morning shifts (M), two afternoon shifts (A) and two night shifts (N). At the end of the cycle, 2 consecutive rest days (R). The result is MM-AA-NN-RR.
4x3 rotation	4 working days followed by 3 rest days.
4x2 rotation	4 consecutive days working in 12 hour-shifts, followed by 2 rest days.
Continuous	All days of the week are covered
Discontinuous	Work does not occur every day of the week, i.e., no work during the weekends
With or without night work	The working time can be extended to all or part of the night (at least 3 hours between midnight and 5:00 AM; between 10:00 PM and 06:00AM in Spain). The number of nights worked per week/month/year can vary considerably according to the speed of shift rotation. Periods of night work can vary:<br>10 hours: from 10:00 PM to 8:00 AM.<br>12 hours: from 7:00 - 8:00 PM to 7:00 - 8:00 AM.
**Characteristics of shift scheduling**	
Duration of shifts	Shift length can range from 6 to 24 hours. The most common length of a shift is 8 hours. 12 hour- and 24 hour-shifts are also common
Speed of shift rotation	Number of consecutive days worked in the same shift
Fast change	Daily, or every 2 or 3 days.<br>It indicates fewer consecutive night shifts than other groups.
Intermediate	Weekly.
Slow	Every 15, 20 or 30 days.
Direction of shift rotation:	The schedule moves to a phase delay or a phase advance rotation.
Forward rotation	Morning/afternoon/night
Backward rotation	Night/afternoon/morning
Length of shift cycle according to a cyclic programming:	Pre-set planning of shifts and breaks distributed equally among staff members.
Short length	Cycles change every 6–9 days.
Intermediate	Cycles change every 20–30 days.
Long	Cycles change every 6 months or more.
Irregular	No cyclic programming.
Correspondence of rest days along the shift cycle and between shifts:	Number and position of rest days/hours between shifts.
Compressed week schedules	The standard work week is reduced to fewer than 5 days by increasing the working hours each day (e.g., four 10-hour days or three 12-hour days)

[26] IARC Working Group on the Evaluation of Carcinogenic Risks to Humans. Painting, firefighting and shiftwork. Vol. 98. Lyon (FR): International Agency for Research on Cancer; 2010.
[27] IARC Working Group on the Identification of Carcinogenic Hazards to Humans. Night shift work. Vol. 124. Lyon (FR): International Agency for Research on Cancer. 2020.
[44] Peplonska B, Bukowska A, Lie JAS, Gromadzinska J, Zienolddiny S. Night shift work and other determinants of estradiol, testosterone, and dehydroepiandrosterone sulfate among middle-aged nurses and midwives. Scand J Work Environ Health. 2016. 42(5):435-446. doi: 10.5271/sjweh.3581
[55] McElvenny DM, Crawford JO, Davis A, Dixon K, Alexander C, Cowie H, Cherrie JW; The Institution of Occupational Safety and Health Board (IOSH). A review of the impact of shift work on occupational cancer. Wigston (UK): IOSH, 2018. Available at: https://www.iosh.co.uk/shiftworkreview (accessed on 31 March 2021).
[70] Directiva 2003/88/CE del Parlamento Europeo y del Consejo, de 4 de noviembre de 2003, relativa a determinados aspectos de la ordenación del tiempo de trabajo. Ref: DOUE-L-2003-81852. Available at: https://www.boe.es/doue/2003/299/L00009-00019.pdf (accessed on 31 March 2021).
