# Supplementary material for: Implications of Lifestyle and Occupational Factors on the Risk of Breast Cancer in Shiftwork Nurses
Source: Healthcare (Basel). 2021 May 30;9(6):649. doi: 10.3390/healthcare9060649 (PMC8228409; doi:10.3390/healthcare9060649)
Supplement: Supplementary file 1 [file healthcare-09-00649-s001.zip › Table S2. Sex analysis.pdf]

Table S2. Sex categorized descriptive analysis.

	N (%)	Female (%) (N=502)	Male (%) (N=56)	χ2	p	Odds Ratio (CI=95%)
Sex						
Breast cancer cases	56 (10.0)	91.1	8.9	.144	.705	1.204
Healthy cases	502 (90.0)	89.4	10.6			(.460, 3.150)
Age						
41 or younger	281 (50.4)	93.6	6.4	9.669	.002	2.466
Older than 41	277 (49.6)	85.6	14.4			(1.376, 4.419)
Marital relationship						
With partner	317 (56.8)	87.7	12.3	2..871	.090	.610
Single	241 (43.2)	92.1	7.9			(.343, 1.085)
Children under 14 years						
Yes	225 (40.3)	88.9	11.1	.208	.648	.880
No	333 (59.7)	90.1	9.9			(.508, 1.525)
Care for dependents at home						
Yes	58 (10.4)	93.1	6.9	.850	.356	1.635
No	500 (89.6)	89.2	10.8			(.570, 4.690)
Mammography (N=497)						
Yes	211 (42.5)	91.9	8.1	2.986	.084	1.696
Never	286 (57.5)	87.1	12.9			(.927, 3.103)
Family history of cancer (N=551)						
Yes	72 (13.1)	93.1	6.9	.850	.356	1.562
No	479 (86.9)	89.6	10.4			(.601, 4.057)
BMI						
Underweight	10 (1.8)	80.0	20.0	2.918	.404	-
Normal	376 (67.4)	91.0	9.0			
Overweight	128 (22.9)	86.7	13.3			
Obese	44 (7.9)	88.6	11.4			
Physical activity at work						
Light	124 (22.2)	91.9	8.1	2.207	.531	-
Moderate	313 (56.1)	89.1	10.9			
Hard	113 (20.3)	87.6	12.4			
Very hard	8 (1.4)	100.0	0			
Physical activity during leisure time						
Two hours or less	286 (51.25)	88.5	11.5	.825	.364	.776
More than 2 hours	272 (28.75)	90.8	9.2			(.448, 1.343)
Tobacco consumption						
Yes	301 (53.9)	89.0	11.0	.227	.634	.875
No	257 (46.1)	90.3	9.7			(.506, 1.515)
Compliance with the smoking ban at work						
Totally	124 (22.2)	94.4	5.6	3.899	.273	-
Almost always	239 (42.8)	88.3	11.7			
Hardly ever	141 (25.3)	87.9	12.1			
Never	54 (9.7)	88.9	11.1			

Exposition to tobacco smoke at home						
More than 5 hours a day	22 (3.9)	77.3	22.7	4.150	.246	-
Between 1 and 5 hours a day	36 (6.5)	88.9	11.1			
Less than 1 hour a day	42 (7.5)	92.9	7.1			
Never or hardly ever	458 (82.1)	90.0	10.0			
Use of medication to sleep						
Yes	116 (20.8)	86.2	13.8	1.816	.178	.656
No	442 (79.2)	90.5	9.5			(.354, 1.215)
Hormone-based oral contraceptives (N=504)						
Yes	334 (66.3)	90.4	9.6	2.950	.086	1.626
Never	170 (33.7)	85.3	14.7			(.930, 2.849)
Shift work at this moment						
No	114 (20.4)	86,8%	13,2%	1.175	.278	.708
Yes	444 (79.6)	90,3%	9,7%			(.378, 1.326)
Night work at this moment						
No	180 (32.3)	86,7%	13,3%	2.464	.116	.642
Yes	378 (67.7)	91,0%	9,0%			(.369, 1.120)
Working experience						
16 years or less	280 (50.2)	90.4	9.6	.341	.559	1.176
More than 16 years	278 (49.8)	88.8	11.2			(.682, 2.028)
Total years performing more than 3 nights a month						
10 years or less	317 (56.8)	90,2%	9,8%	.298	.585	1.164
More than 10 years	241 (43.2)	88,8%	11,2%			(.675, 2.008)
Total worked nights						
Less than 500 night	265 (47.5)	89.4	10.6	.016	.899	1.036
500 night or more	293 (52.5)	89.8	10.2			(.601, 1.785)
Total sick leaves over lifespan (N=550)						
2 or less	342 (62.2)	89.8	10.2	.093	.760	1.091
More than 2	208 (37.8)	88.9	11.1			(.625, 1.903)
Sick leaves in the last year (N=554)						
Without sick leave	385 (69.5)	90.1	9.9	.240	.624	1.157
With sick leave	169 (30.5)	88.8	11.2			(.646, 2.072)
